# A novel biallelic *LMNB2* variant in a patient with progressive myoclonus epilepsy and ataxia: A case of laminopathy

**DOI:** 10.1002/ccr3.4520

**Published:** 2021-08-23

**Authors:** Saeed Farajzadeh Valilou, Javad Karimzad Hagh, Mohammad Salimi Asl, Isa Abdi Rad, Masoud Edizadeh, Arash Pooladi

**Affiliations:** ^1^ Department of Medical Genetics and Sarem Cell Research Center (SCRC) Sarem Womens' Hospital Tehran Iran; ^2^ Medical Genetics Network (MeGeNe) Universal Scientific Education and Research Network (USERN) Tehran Iran; ^3^ IVF Center Cuypers & Cuypers Fertility Center Heinsberger Höfe Heinsberg Germany; ^4^ Departament of Medical Genetics School of Medicine Urmia University of Medical Sciences Urmia Iran; ^5^ Biotechnology Department School of Medicine Lorestan University of Medical Sciences Khorramabad Lorestan Iran; ^6^ Cancer and Immunology Research Center Research Institute for Health Development Kurdistan University of Medical Sciences Sanandaj Iran; ^7^ Department of Medical Genetics Faculty of Medicine Kurdistan University of Medical Sciences Sanandaj Iran

**Keywords:** ataxia, laminopathy, *LMNB2*, progressive myoclonus epilepsies, whole‐exome sequencing

## Abstract

The report of LMNB2‐related progressive myoclonus epilepsy and ataxia due to missense homozygous c.473G>T variant.

## Graphical abstract

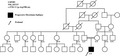

## 1 INTRODUCTION

We report a patient presenting autosomal recessive progressive myoclonus epilepsy. Whole‐exome sequencing was used, and a novel missense homozygous c.473G>T variant in the *LMNB2* gene was identified in the proband. The co‐segregation study confirmed the pathogenic effect of the finding in the family.

Progressive myoclonus epilepsies (PMEs) are a rare group of disorders that present with action myoclonus, tonic‐clonic seizures, and progressive neurological deterioration, typically with cerebellar signs and dementia. These are disorders of gray matter, and neuroimaging reveals cerebral and cerebellar atrophy and no white matter disease. Most molecularly characterized PMEs are inherited in an autosomal recessive manner, with rare cases showing autosomal dominant or mitochondrial inheritance.^1^, ^2^ There are five main causes of PME comprising (1) Unverricht‐Lundborg disease due to CSTB gene mutations; (2) Lafora disease caused by EPM2A and NHLRC1 alterations; (3) myoclonic epilepsy with ragged red fibers caused by MTTK variants; (4) neuronal ceroid lipofuscinosis as a result of a mutation in TPP1, CLN3, CLN5, and CLN6; and (5) sialidoses as a consequence of neuraminidase deficiency in leukocytes or fibroblasts that have been more accurately defined with recent advances in genetic studies.^1^, ^3^, ^4^ PME‐associated genes encode a variety of proteins, many of which are associated with the endosomal and lysosomal function ^5^; however, the association of *LMNB2*, a lamin‐coding gene, with PME has not been reported until a variant was identified in two Palestinian patients diagnosed with PME in 2015.^6^

Herein, we report a family with an affected boy who presented myoclonic seizure from consanguineous parents and exhibiting an autosomal recessive pattern of inheritance. Whole‐exome sequencing was performed and identified a homozygous missense variant in the *LMNB2* gene that encodes lamin B2, one of the major structural proteins in the nuclear filament. Although *LMNA*, another gene in the lamin family, has been reported with particular diseases such as cardiomyopathy, muscular dystrophy, lipodystrophy, and progeroid syndromes,^7^ *LMNB2* has been reported to cause lipodystrophy,^8^ and its pathogenicity in developing disease has not been studied well.

## 2 CLINICAL REPORT

A 5‐year‐old boy was referred to our clinical genetic department because of progressive wide‐based ataxic gait and intractable myoclonic seizure. Following an uneventful pregnancy, he was born at 38 weeks of gestation via cesarean section to healthy consanguineous Iranian parents with a high inbreeding coefficient (F = 9.375%). His anthropometric features and Apgar score were normal at birth, and dysmorphology was unremarkable. Psychomotor development was normal until 15 months of age when he manifests unsteady gait with frequent stumbling. After three months, at age 18 months, he revealed myoclonic seizure of limbs during sleeping and awake, which evolve to intractable generalized myoclonic seizure over time.

At age three years, his brain MRI, nerve conduction velocity (NCV), electromyography (EMG), and metabolic survey were normal, where EEG was significant for generalized epileptic discharge. Initially, some improvement in myoclonic seizure was observed by using Synacthen, but seizure became worse over time even after adding multiple drugs including IVIg, pulse therapy of prednisolone, profilin, and the ketogenic diet. At age five years, in addition to his multi‐AED intractable seizure, he developed progressive ataxia, intension tremor, and slurred speech.

## 3 MATERIALS AND METHODS

### 3.1 Patient recruitment and sampling

This study was approved by the Research Committee and Department of Molecular Genetics of Sarem Women Hospital, Tehran, Iran. We obtained informed consent, clinical data, and pedigree information from the family. Peripheral whole blood was obtained from the proband and the family. Subsequently, genomic DNA was extracted using salting‐out method.^9^ Then, whole‐exome sequencing (WES) was performed on the proband's DNA, and data were interpreted and analyzed.

### 3.2 WES and Variant prioritization

Genomic DNA was isolated from white blood cells and subjected to exome capturing using Agilent SureSelect (Santa Clara, CA), followed by next‐generation sequencing on the Illumina sequencing platform HiSeq 4000 (San Diego, CA), with coverage of >80‐100X. Sequence reads were mapped to the human reference genome (GRCh37/hg19) using BWA program. Variant calling was performed using a Picard and GATK‐Lite toolkit (Broad Institute, Cambridge, MA). Gene annotation of variants in the generated VCF file was performed using wANNOVAR software (http://wannovar.wglab.org/). The generated file underwent filtering variants based on inheritance patterns of the disease in the pedigree. In the second step, synonymous variants and nonexonic variants that were not located within splice site regions were excluded. Then, variants with minor allele frequencies greater than 1% in 1000Genome, ExAC, and GnomAD were filtered out. Following that, remaining variants were filtered based on genotype‐phenotype correlation in the proband using OMIM, GHR, GeneReviews, and Orphanet databases and publications. In step 5, remaining variants were ranked based on their probable impact on protein sequence and function, considering evolutionary conservation among orthologs across phylogeny and in silico prediction programs (such as SIFT, PolyPhen‐2, MutationTaster, CADD, GERP++, and DANN). Next, remaining variants were prioritized upon clinical features of the proband and further evaluated by reviewing the existing literature. The candidate variants were confirmed in the patient's original DNA by Sanger sequencing using primers of forward (5′‐CTCGGTGACCTTGTCTTTGG‐3′) and reverse (5′‐GAGGGTGGACTTTGCGTTTC‐3′).

## 4 RESULTS

**FIGURE 1:**
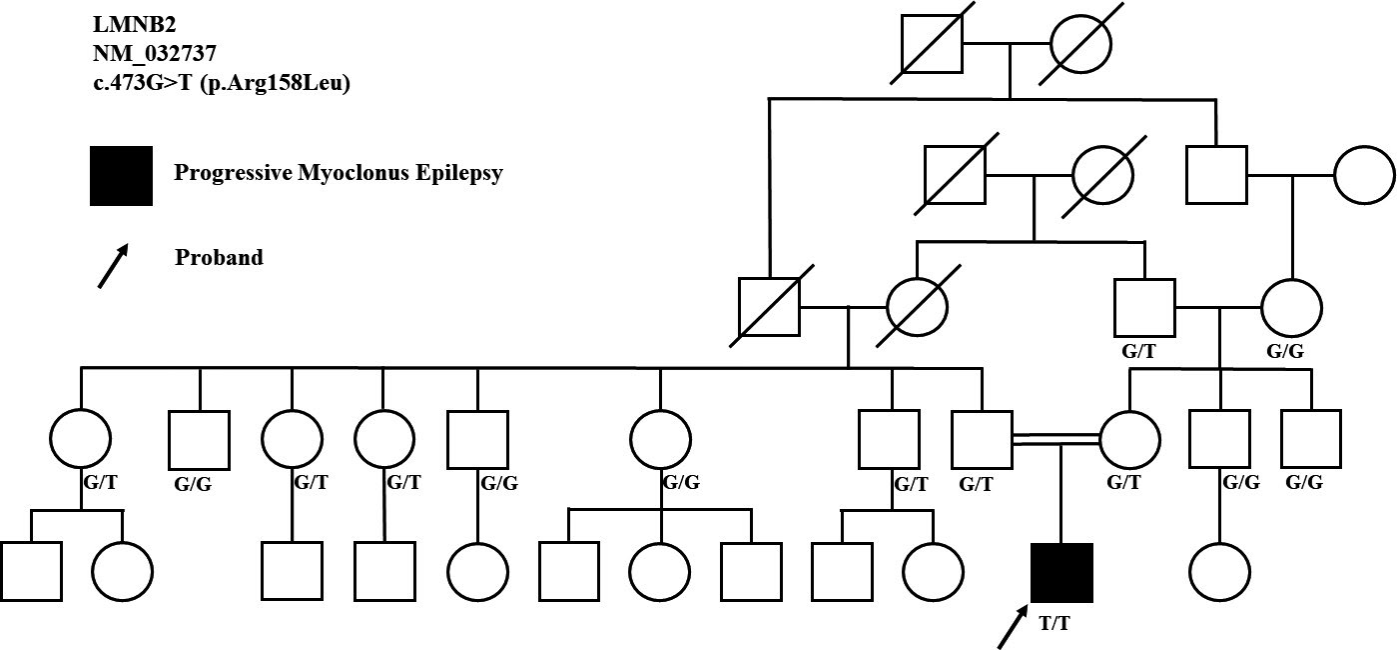
Pedigree of the family with consanguineous marriage and an autosomal recessive pattern of inheritance. The proband is indicated by an arrow. Segregation analysis of the proband and family for the c.473G>T variant and genotypes of thirteen persons are depicted in the figure

**FIGURE 2:**
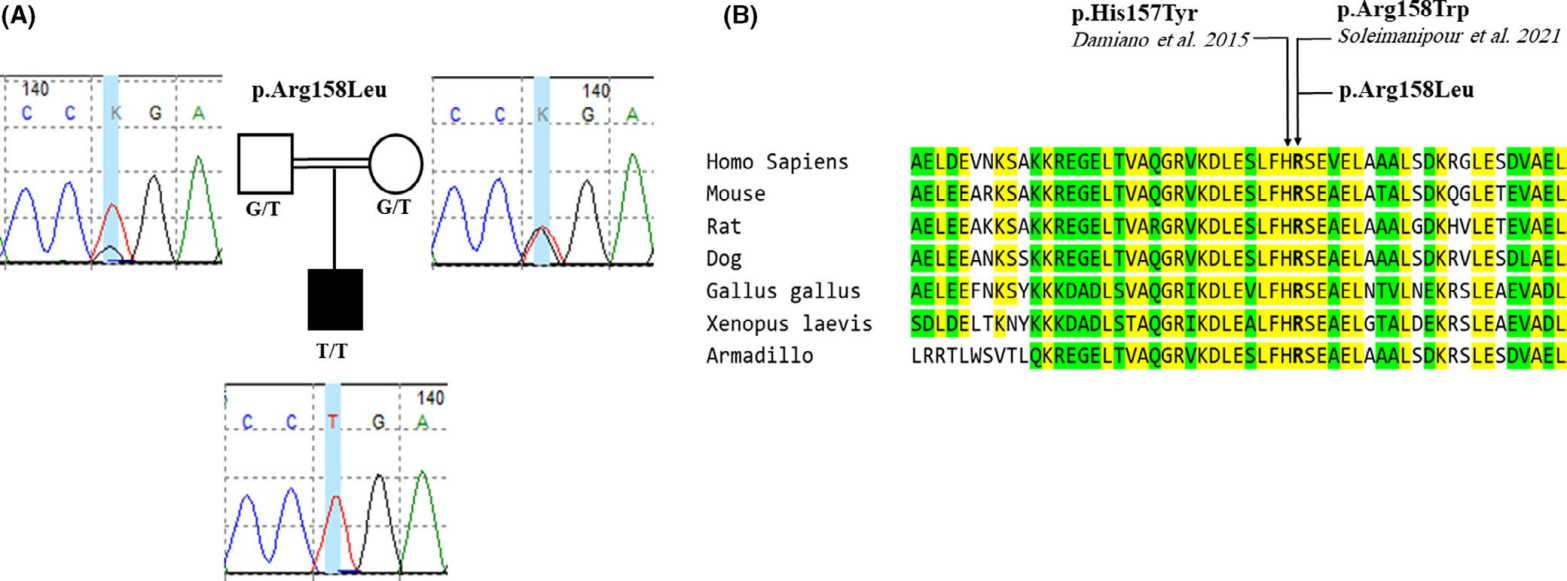
(A) Sequencing electropherograms of c.473G>T variant the proband and the parents. Electropherograms depicting the homozygosity for the c.473G>T variant and are compatible with the patient's phenotype. The parents harbor the heterozygote alleles. (B) Amino acid sequence alignment at the position of p. Arg158 in seven different species Human (Homo Sapiens, accession NM_032737.4), Mouse (Mus musculus, accession NM_010722.5), Rat (Rattus norvegicus, accession NM_001305235.1), Dog (Canis lupus, accession XM_014121833.2), chicken (Gallus gallus, accession NM_205285.1), Xenopus laevis (accession NM_001087478.1) and Armadillo (Dasypus novemcinctus, accession XM_023591881.1). Identical amino acids are highlighted in yellow, positions where two amino acids are identical are highlighted in green. This alignment presents a reasonably conserve region in the seven species. In addition, the p. Arg158 and p. His157 (Damiano et. al 2015^6^) showing highly conserved residues among sp.

A novel biallelic nonsynonymous missense variant NM_032737 c.473G>T (p. Arg158Leu) was identified in the *LMNB2* gene that is classified as pathogenic based on ACMG guidelines. Following WES, Sanger sequencing was carried out for co‐segregation analysis on the proband and the family members. The variant was confirmed in the proband, and both of the parents were heterozygote for the c.473G>T variant. Further co‐segregation analysis revealed that the grandmother, but not grandfather, from the mother side, and four of the father's siblings (13 persons in the families) were carriers (Figure 1 and Figure 2A).

**FIGURE 3:**
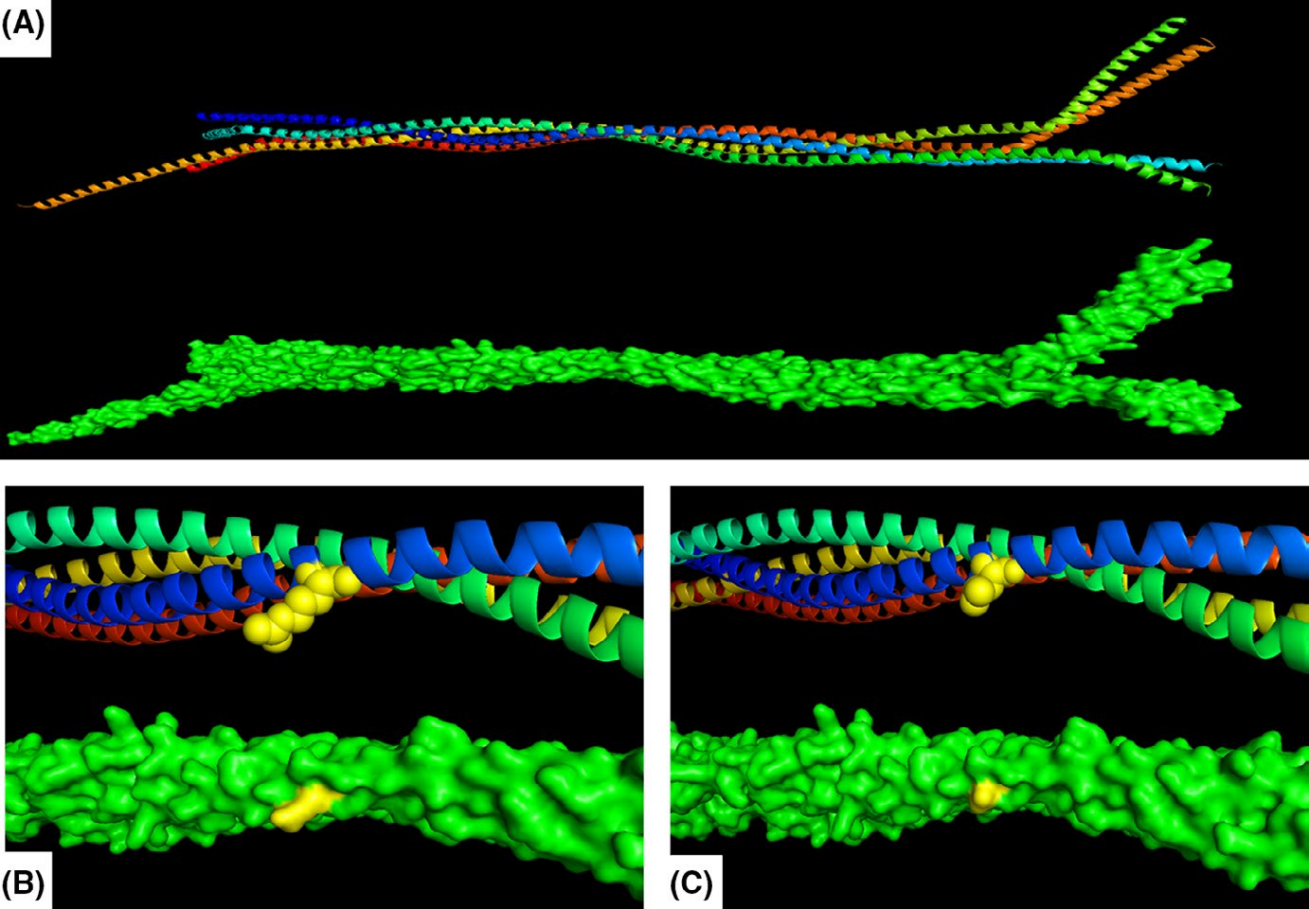
LMNB2 protein 3D model. (A) Protein structure of LMNB2 comprised of four alpha helices which in blue, green, yellow and red. (B) The position of the Arginine amino acid in the domain. (C) Amino acid change of Arginine to Leucine as shown in the 3D model. Arginine is a positively charged basic amino acid. Substitution of p. Arg158 with Leucine, a branched chain non‐polar amino acid with hydrophobic side chain may affect the conformation of the alpha helix and sabotage the domain 1B of LMNB2. The protein 3D model was obtained from “https://swissmodel.expasy.org/repository/uniprot”. The ID for the protein model is 6jlb.1.A

The c.473G>T variant is predicted as a damaging and disease‐causing variant according to in silico predictors such as SIFT, MutationTaster, and CADD. Also, the c.473G>T variant is ultra‐rare (1x, allele frequency: 0.000004038) in GnomAD. In addition, bioinformatics study of the Arg158 and nearby regions exhibited high conservation among the species (Figure 2‐B). Protein 3D modeling was carried out to assess a possible protein alteration (Figure 3). Although the alteration result was insignificant, considering the type of amino acid change from arginine—a basic amino acid with a positively charged side chain—to leucine, a nonpolar amino acid, it is highly probable that it can affect the conformation of the protein helix. In other words, the positively charged side chain of arginine is lost with the leucine substitution.

## 5 DISCUSSION

**FIGURE 4:**
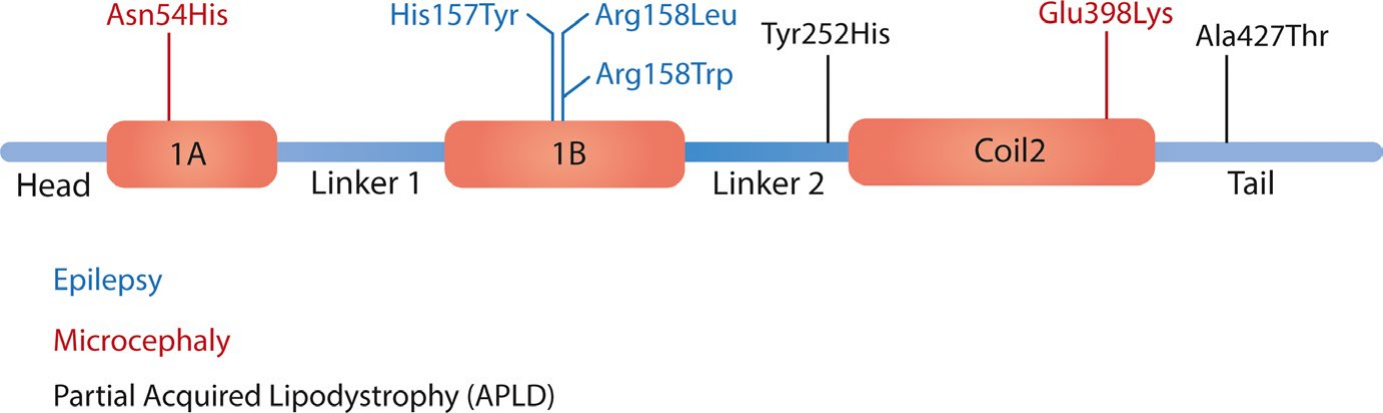
This figure shows the domains of lmnb2 protein as well as the position of detected amino acid changes. EMP9 associated amino acid alterations including His157Tyr, Arg158Leu and Arg158Trp are located in 1B domain and highlighted by blue. Similarly, variants which cause primary microcephaly are located in coiled coil domains. In contrast, APLD associated alterations occurred outside of these critical domains

In the current study, we described an Iranian affected offspring with indications of progressive wide‐based ataxic gait and intractable myoclonic seizure with a homozygous missense c.473G>T variant in the *LMNB2* gene. The *LMNB2* gene encodes an intermediate nuclear filament, which together with other components of the nuclear lamina provides a scaffolding meshwork. So far, genetic alterations in the *LMNB2* gene have been associated with three distinct phenotypes, such as partial acquired lipodystrophy (APLD),^8^ epilepsy, progressive myoclonic 9 (EPM9),^6^ and recently found autosomal dominant primary microcephaly.^10^ Interestingly, few heterozygote variants including (NM_032737.4):c.1279G>A and (NM_032737.4):c.754T>C in the tail and linker region of the lmnb2 protein have been classified as risk factors for acquired lipodystrophy (Figure 4).^8^ Individuals with *LMNB2*‐associated APLD represented progressive lipodystrophy in different parts of the body at various ages. However, there was not any sign of lipodystrophy neither in the proband nor in his carrier parents during the examination at the time of the study. It is not surprising because lamin gene alterations only affect specific types of tissues, as they have been associated with various disorders of the nervous system.^7^, ^11^, ^12^ This tissue specificity might explain why skin keratinocytes of Lmnb1 and Lmnb2 knockout mouse are able to proliferate normally in addition to embryonic stem cells of B‐type lamin knockout mouse, which are viable and self‐renewable.^13^, ^14^ In contrast, lmnb2‐deficient mouse dies immediately after birth because of neuronal layering defect in the cerebral cortex following interrupted neuronal migration from the ventricular zone to the cortical plate.^15^ Apart from this ambiguous role of LMNB2 in lipid tissue disorders, it is obvious that LMNB 1/2 is involved in cerebral cortex development and function. Although lamins B1 and B2 have almost 60% sequence similarity at the amino acid level, it seems that each plays unique and specific role in the earliest stages of neuronal cell development and later in its maintenance.^16^ In fact, lamin B2 deficiency is not compensated by increased lamin b1 synthesis, and the disease phenotype would elicit. Recently, two heterozygote variants in the *LMNB2* gene have been reported as a genetic cause of primary microcephaly and intellectual disability.^10^ Table 1 compares the phenotype findings of the proband with the previously reported EPM9 patients. The proband's phenotype is compatible with previously described EPM9 patients, except in severity and some signs and symptoms. In fact, the age of onset in the proband is significantly earlier than affected individuals in Damiano JA et al's study, at age 1 year compared to 6–7 years, respectively. This finding suggests that the novel variant can associate with more severe phenotype, at least in this case.

**TABLE 1:** PME patients with *LMNB2* variants

	Patient in this publication	Damiano JA et al. (2015)^6^		Soleimanipour et al. (2021)
		Patient 1	Patient 2	
Gender	Male	Female	Female	Female
Age at the time of publication	5 years	Not alive (Died at 20 years due to status epilepticus)	12 years	10 years
Mutation	Missense	Missense	Missense	Missense
Variant	c.473G>T (p. Arg158Leu)	c.469C>T (p. His157Tyr)	c.469C>T (p. His157Tyr)	c.472C > T (p. Arg158Trp)
Inheritance pattern	Recessive	Recessive	Recessive	Recessive
Age of onset	1 year	7 years	6 years	1 year
At birth	Normal Apgar	–	–	Full term Normal
Mouth	–	–	Small tongue	–
Neurologic	Ataxic gait Frequent stumbling Myoclonic seizure of limbs during sleeping and awake Intractable myoclonic seizure Progressive ataxia Intension tremor Slurred speech	Cognition was normal. Gait deteriorated Frequent falls Seizures with falling Tonic‐clonic seizures (age 9 years) Seizures became resistant to anti‐epileptic drug Severe action myoclonus involving limbs and bulbar muscles Worsening epilepsy	Developmental delay First talked at 20 months Walked at 2 years Special school Intellect was low Gait deteriorated with seizure onset in her seventh year Morning myoclonus Tonic‐clonic seizures at 6.5 years	Limb shivering and intermittent jerky movements Frequent seizures resulted in neurologic regression Loss of speech at 6 years No sit or walk at 7 years Severe truncal hypotonia Horizontal nystagmus. Normal cognition Worsening seizure episodes
Muscle and soft tissue	‐	Diffuse muscle wasting Diffuse loss of subcutaneous fat	Wasted with little subcutaneous fat	Symmetric decreased deep tendon reflexes in four limbs Hypotonia
Skeletal	Normal	Scoliosis	Scoliosis Short thumbs and curved digits	‐
MRI, NCV, EMG, and EEG	Normal brain MRI, NCV, and EMG Significant for generalized epileptic discharge in EEG test	MRI at 9 years was normal	MRI at 18 months showed complete agenesis of the corpus callosum, venticulomegaly, and a left‐sided interhemispheric cyst	Focal onset spike waves in EEG Generalized high‐voltage sharp waves and multifocal spikes and waves in video EEG Myopathic pattern in four limbs in EMG Moderate cerebral and cerebellar atrophy in MRI at 7 years Progressive generalized atrophy in MRI at 8 years
Laboratory abnormalities	–	–	–	Normal serum ammonia and lactate, urine organic acids profile, acylcarnitine profile, and cerebrospinal fluid amino acid chromatography

For the first time, the His157Tyr mutation in the *LMNB2* gene has been related to the progressive myoclonic epilepsy (PME).^6^ In order to understand whether this mutation disrupts lamin b2 regular fibrillar formation or not, Damiano JA et al. compared wild‐type and His157Tyr mutant lamin b2 by fibrillar assembly study. In conclusion, wild‐type lamin b2 formed regular and extended “spikes or needles” with 24.5‐nm repeat pattern. Lateral fiber arrays connected to the main spike on a regular and well‐organized manner and formed extended so‐called paracrystalline arrays. However, the mutant lamin b2 formed irregular and unorganized network‐type structures and 24.5‐nm repeat pattern was absent in the major segments and only confined to partial parts of the structures. Moreover, the His157Tyr mutation, similar to wild‐type protein, did not disrupt the longitudinal assembly since it is located in a coiled coil 1b domain, instead of the a‐helical central rod domain, which mediates a head‐tail overlap in the longitudinal process. These findings suggesting that the coiled coil 1b domain is necessary for regular and organized lateral connections of fiber arrays in the nuclear lamina.^6^ Recently, a novel variant Arg158Trp (c.472C>T) at the same amino acid codon with only one base pair difference in the cDNA position with a c.473G>T variant is identified ^17^ and strengthens the association of LMNB2 and PME9. Our novel candidate variant, Arg158Leu (c.473G>T), together with the previously reported pathogenic variants His157Tyr ^6^ and Arg158Trp ^17^ are located side by side in the highly conserved coiled coil 1b domain of lamin b2 protein. The presence of these two amino acids close to each other at the protein level and similar clinical outcomes of disrupted His157 and Arg158 amino acids highlight the fact that Arg158Leu mutation might affect fibrillar assembly in a similar way by disrupting regular fiber formation.

Lamin‐containing protein meshwork, like a main scaffold of a bridge, connects to a bunch of proteins including nuclear pore complexes (NPCs), SUN domain proteins, and additional nuclear envelope transmembrane proteins, and makes a physical link between nucleoskeleton and cytoskeleton that helps the nuclear envelope sustain its proper shape and integrity.^18^, ^19^ In addition, the nuclear lamina plays a role in housekeeping functions as it suppresses expression of various genes in two different ways. First, it directly binds to a vast majority of genes in human cells, including nuclear lamina–associated genes, and makes them transcriptionally inactive.^20^, ^21^ Interestingly, depressed lamin‐associated genes have been observed in lamin b2 knockout Drosophila, indicating the role of lamin b2 in expression controlling of the series of genes.^21^, ^22^ Second, the nuclear lamina acts as an anchoring point for chromatin and organize the global three‐dimensional genome.^23^, ^24^ Chromatin detachment from the nuclear lamina due to lamin mutations can lead to the chromatin relaxation and subsequent gene expression.^25^ Despite its role in sustaining nuclear shape and expression controlling of several genes, lamin b2 plays an extra nuclear role by localizing to mitochondria in axons.^26^ Developing axons require a significant amount of energy to reach their distinct synaptic targets during wiring of the central nervous system. It has been demonstrated that lamin b2 is transported to mitochondria and maintain mitochondrial function to provide energy for the cell.^26^ Therefore, it is required in developing axons and their subsequent maintenance. In addition to axons, LMNB2 is strongly expressed throughout oligodendrocyte developing process and plays a role in developing and maintaining of this kind of glial cells.^27^ Several studies have illustrated a critical role of lamin b2 in the nervous system as it involves in neuronal migration and subsequent survival and function.

In summary, our findings confirm the genotype‐phenotype correlation between the LMNB2 gene and EMP9 as a third case worldwide and suggests Arg158Leu as a pathogenic variant. However, functional studies are strongly recommended for confirmation of the true pathogenic effect of the variant prior to any genetic counseling or medical intervention.

## CONFLICT OF INTEREST

None declared.

## AUTHOR CONTRIBUTIONS

SFV, JKH, and AP: study design; SFV and ME: manuscript preparation; SFV, IAR, and AP: critically reviewed and revised the manuscript; SFV and MSA: data analysis and data collection; JKH: patient recruitment; SFV: laboratory work; IAR and AP: genetic counseling and clinical examination; and AP: funding and supervision of the research.

## ETHICAL APPROVAL

Prior to the study, written consent was obtained from the patient's parents. This research was approved and licensed by the Kurdistan University of Medical Sciences with the code of ethics IR.MUK.REC.1400.072.

## Data Availability

The authors confirm that the all data relevant to the findings of this study are included in the article.
